# Impact of the Saving Mothers, Giving Life Approach on Decreasing Maternal and Perinatal Deaths in Uganda and Zambia

**DOI:** 10.9745/GHSP-D-18-00428

**Published:** 2019-03-11

**Authors:** Florina Serbanescu, Thomas A. Clark, Mary M. Goodwin, Lisa J. Nelson, Mary Adetinuke Boyd, Adeodata R. Kekitiinwa, Frank Kaharuza, Brenda Picho, Diane Morof, Curtis Blanton, Maybin Mumba, Patrick Komakech, Fernando Carlosama, Michelle M. Schmitz, Claudia Morrissey Conlon

**Affiliations:** a Division of Reproductive Health, U.S. Centers for Disease Control and Prevention, Atlanta, GA, USA.; b Division of Global HIV and TB, U.S. Centers for Disease Control and Prevention, Kampala, Uganda.; c Division of Global HIV and TB, U.S. Centers for Disease Control and Prevention, Lusaka, Zambia.; d Baylor College of Medicine Children's Foundation-Uganda, Kampala, Uganda.; e HIV Health Office, U.S. Agency for International Development, Kampala, Uganda.; f Infectious Diseases Institute, College of Health Sciences, Makerere University, Kampala, Uganda.; g U.S. Public Health Service Commissioned Corps, Rockville, MD, USA.; h Bureau for Global Health, U.S. Agency for International Development, Washington, DC, USA.

## Abstract

**Background:**

Maternal and perinatal mortality is a global development priority that continues to present major challenges in sub-Saharan Africa. Saving Mothers, Giving Life (SMGL) was a multipartner initiative implemented from 2012 to 2017 with the goal of improving maternal and perinatal health in high-mortality settings. The initiative accomplished this by reducing delays to timely and appropriate obstetric care through the introduction and support of community and facility evidence-based and district-wide health systems strengthening interventions.

**Methods:**

SMGL-designated pilot districts in Uganda and Zambia documented baseline and endline maternal and perinatal health outcomes using multiple approaches. These included health facility assessments, pregnancy outcome monitoring, enhanced maternal mortality detection in facilities, and district population-based identification and investigation of maternal deaths in communities.

**Results:**

Over the course of the 5-year SMGL initiative, population-based estimates documented a 44% reduction in the SMGL-supported district-wide maternal mortality ratio (MMR) in Uganda (from 452 to 255 maternal deaths per 100,000 live births) and a 41% reduction in Zambia (from 480 to 284 maternal deaths per 100,000 live births). The MMR in SMGL-supported health facilities declined by 44% in Uganda and by 38% in Zambia. The institutional delivery rate increased by 47% in Uganda (from 45.5% to 66.8% of district births) and by 44% in Zambia (from 62.6% to 90.2% of district births). The number of facilities providing emergency obstetric and newborn care (EmONC) rose from 10 to 26 in Uganda and from 7 to 13 in Zambia, and lower- and mid-level facilities increased the number of EmONC signal functions performed. Cesarean delivery rates increased by more than 70% in both countries, reaching 9% and 5% of all births in Uganda and Zambia districts, respectively. Maternal deaths in facilities due to obstetric hemorrhage declined by 42% in Uganda and 65% in Zambia. Overall, perinatal mortality rates declined, largely due to reductions in stillbirths in both countries; however, no statistically significant changes were found in predischarge neonatal death rates in predischarge either country.

**Conclusions:**

MMRs fell significantly in Uganda and Zambia following the introduction of the SMGL interventions, and SMGL's comprehensive district systems-strengthening approach successfully improved coverage and quality of care for mothers and newborns. The lessons learned from the initiative can inform policy makers and program managers in other low- and middle-income settings where similar approaches could be used to rapidly reduce preventable maternal and newborn deaths.

## Abstract (precis)

Through district system strengthening, integrated services, and community engagement interventions, the Saving Mothers, Giving Life initiative increased emergency obstetric care coverage and access to, and demand for, improved quality of care that led to rapid declines in district maternal and perinatal mortality. Significant reductions in intrapartum stillbirth rate and maternal mortality ratios around the time of birth attest to the success of the initiative.

## INTRODUCTION

Globally, more than 300,000 maternal deaths due to complications of pregnancy and childbirth occurred in 2015, 201,000 of which occurred in sub-Saharan Africa.^1^ Additionally, of the approximately 2.7 million neonatal deaths that occurred and 2.6 million babies who were stillborn in 2015, about 1 million neonatal deaths^2^ and 1 million stillbirths occurred in sub-Saharan Africa.^3^^,^^4^ Roughly, 30% of neonatal deaths and 50% of the stillbirths in sub-Saharan Africa were due to intrapartum complications.^2^^–^^4^ Reductions in maternal and neonatal mortality and stillbirths have been prioritized in the United Nations Sustainable Development Goals (SDGs) 3.1 and 3.2 that promote targets of fewer than 70 maternal deaths per 100,000 live births, 12 or fewer neonatal deaths per 1,000 live births, and 12 or fewer stillbirths per 1,000 births by 2030.^5^ These targets are echoed in the updated World Health Organization (WHO) Global Strategy for Women's, Children's and Adolescents' Health (2016–2030) to advance progress toward reaching the SDGs.^6^

Despite an annual reduction of 2.5% per year from 1990 to 2015, the maternal mortality ratio (MMR) of 546 maternal deaths per 100,000 in sub-Saharan Africa remains the highest regional MMR in the world.^1^ Similarly, the neonatal mortality rate of 28 neonatal deaths per 1,000 live births is the highest globally and its 2% annual decline rate is the lowest.^7^ A large proportion of women and infants die because they do not receive appropriate routine care and do not have support to address the “3 delays”: (1) delayed recognition of a pregnancy complication and decision to go to a facility, (2) delays in reaching an emergency obstetric care facility, and (3) delays in receiving adequate and appropriate obstetric and neonatal care at a health care facility.^8^

Maternal and neonatal deaths at the time of delivery and postpartum are largely preventable using the 9 evidence-based lifesaving interventions, called “signal functions,” which comprise emergency obstetric and neonatal care (EmONC) services.^9^ Basic EmONC (BEmONC) facilities provide 7 of the signal functions: (1) administer parenteral antibiotics, (2) administer uterotonic drugs for active management of the third stage of labor and prevention of postpartum hemorrhage, (3) use parenteral anticonvulsants for the management of preeclampsia/eclampsia, (4) perform manual removal of placenta, (5) perform removal of retained products, (6) perform assisted vaginal delivery, and (7) perform basic neonatal resuscitation. Comprehensive EmONC (CEmONC) facilities perform the 7 basic signal functions, plus 2 more: cesarean delivery and blood transfusion.^10^ Access to EmONC remains a global challenge, with only 1 in 5 pregnant women experiencing pregnancy complications receiving emergency obstetric care.^11^

Access to EmONC remains a global challenge, with only 20% of pregnant women experiencing pregnancy complications receiving emergency obstetric care.

Since obstetric complications are often unpredictable, WHO proposed a 2-hour travel time to the nearest facility with surgical capacity as the benchmark of access; 2 hours is the estimated interval from onset of bleeding to death if a woman with obstetric hemorrhage does not receive adequate treatment.^10^^,^^12^ More broadly, experts have recommended that at least 80% of the entire population should have access to emergency surgical care within 2 hours.^13^

Saving Mothers, Giving Life (SMGL) was a multipartner initiative designed to reduce deaths stemming from complications of pregnancy and childbirth through proven interventions that increase access to, use of, and quality of facility delivery and EmONC services, including improved newborn care.^14^ SMGL simultaneously implemented multiple interventions to target the 3 delays by applying a comprehensive approach to strengthen district health systems (Table 1). SMGL sought to ensure that every pregnant woman is aware of the benefits of facility-based care and has access to, and uses, quality obstetric services. The initiative established an ambitious target of achieving a 50% decline in the MMR in the pilot districts to accelerate progress toward global goals and commitments.

**TABLE 1.** SMGL Strategies and Interventions Implemented in Uganda and Zambia to Reduce the 3 Delays, 2012–2017

Strategy	Approach/Intervention	Primary DelayAddressed^a^
Promote community engagement and empowerment for improved maternal and newborn health	Implement community-based communication and education messages on safe motherhood via mass media and community events, which includes displaying SMGL messages in public places to promote safe motherhood, broadcasting radio messages and programs, developing a documentary in Zambia, and supporting local drama groups in performing skits and traditional songs Build stronger partnerships between communities and facilities, which includes supervision and support provided by facility health workers to community volunteers Engage communities in monitoring and evaluation, which includes participation of VHTs in the SMGL baseline and endline evaluation of population maternal mortality ratio and MDSR (Uganda)	1
Increase birth preparedness, demand for facility delivery, and use of preventive health care services	Assist with community activities aimed to increase birth preparedness, knowledge of pregnancy danger signs, and use of antenatal care, facility-based delivery, and postnatal care services Extend the delivery system of preventive services by using mobile and community outreach clinics to provide antenatal care, HIV counseling and testing, immunization, and postpartum family planning; ensuring provision of postpartum home care for mothers and newborns; distributing commodities through Mama Ambassadors (Uganda); and distributing birth plans through community volunteers and change champions (Zambia)	1
Decrease financial and logistic barriers to accessing facility delivery care	Market and distribute clean delivery kits Market and distribute transport vouchers to subsidize access to facility delivery, antenatal, and postnatal care services Promote community-based loans to increase use of facility delivery care services	1 and 2
Decrease distance to facility-based delivery services by increasing the number of EmONC facilities	Establish additional EmONC facilities and strengthen existing ones to provide: clean and safe basic delivery services; quality HIV counseling and testing; management of routine and complicated deliveries; essential and specialized newborn care; and timely referrals Implement interventions to improve facility renovations, including building operation theaters and maternity waiting homes; expanding/upgrading maternity wards, neonatal special care units, and laboratories and pharmacies; purchasing equipment, supplies, and essential medicines; and hiring and training nurses, midwives, doctors, and anesthetists in EmONC	2 and 3
Improve the accessibility of EmONC facilities	Create a 24 hour a day/7 day a week communication/transportation system that is consultative, protocol-driven, quality-assured, and integrated (public and private) to ensure that women with complications reach emergency services within 2 hours Implement interventions such as purchasing ambulances and other motorized vehicles; supporting operating costs of transport, such as maintenance, insurance, and petrol; setting up district transportation committees to improve coordination of ambulances; and renovating and building maternity waiting homes	**2**
Ensure facilities providing delivery care have adequate infrastructure	Support uninterrupted access to electricity and water Implement interventions such as procuring solar panels and generators and ensuring safe water systems in maternity wards (water tanks and provision of piped water) Support expansions, renovations, and facility enhancements to accommodate additional deliveries (including renovating and building operation theaters, expanding labor rooms, and adding postpartum wards) Support facility enhancements to improve neonatal survival, including renovating infrastructure to provide space for KMC and neonatal special care units and procuring special equipment (incubators, infant warmers, and phototherapy lamps)	3
Ensure sufficient medical supplies, equipment, and essential medicines	Strengthen supply chains for essential supplies and medicines Strengthen availability of blood supplies and surgical equipment, including the opening of new blood banks	3
Ensure sufficient well-trained health care providers at facilities	Recruit new medical doctors and nurse-midwives through a joint hiring process with the districts Conduct trainings and refresher courses including: basic EmONC trainings, surgical skills course for medical officers, management of postpartum hemorrhage using uterine balloon tamponade, essential newborn care and neonatal resuscitation, and KMC Provide mentoring and supportive supervision to newly hired and existing personnel	3
Improve quality of care and ensure care is evidence-based	Implement quality effective interventions, such as partograph use, active management of the third stage of labor, KMC, improved infection control practices, and management of obstetric complications protocols to prevent and treat obstetric and newborn complications Ensure reliable delivery of quality essential and emergency maternal and newborn care, which includes interventions such as the training of midwives in respectful maternity care and the use of facility-generated data to review quality of care and implement practice changes Develop guidelines and policies, and ensure protocol adherence through activities such as the introduction of clinical guidelines and protocols for diagnosing and managing most common obstetric emergencies, delivery checklists, and a tool to prevent perinatal deaths by using data to guide actions (BABIES matrix)	3
Ensure referral capacity exists to support transfers to higher level of care	Improve referral communication systems through increased communication capacity and introduction of referral protocols and forms Ensure timely referrals through purchase of motorized vehicles, support of operating costs of transport, and promotion of district-level coordination	3
Strengthen health management information system and maternal and perinatal death surveillance	Set up pregnancy outcomes monitoring surveillance in health facilities and train health providers and health monitoring officers in data recording, data abstraction, data entry, and data file management Strengthen maternal and perinatal death surveillance in health facilities, including the development of national standards for MDSR Train medical doctors in assigning causes of maternal death using ICD-MM Train health personnel in conducting maternal and perinatal death reviews at facility and district levels Introduce a community MDSR system using the VHTs and other district personnel and develop protocols and tools, including an electronic data monitoring system	1,2,3

Abbreviations: BABIES, birthweight group age-at-death boxes for an intervention and evaluation system; EmONC, emergency obstetric and newborn care; ICD-MM, International Classification of Diseases–Maternal Mortality; KMC, kangaroo mother care; MDSR, maternal death surveillance and response; SMGL, Saving Mothers, Giving Life; VHTs, village health teams.

Note: Detailed information about SMGL country-specific interventions targeting each of the 3 delays are included elsewhere in this supplement.

a Primary delay addressed refers to which of the 3 delays the interventions are assumed to primarily address, since some of the interventions may address more than one delay. 1=First Delay; 2=Second Delay; 3=Third Delay.

SMGL focused on a district health systems-strengthening strategy that was implemented in close collaboration with the national, district, and local governments of Uganda and Zambia and implementing partners, which included the U.S. Centers for Disease Control and Prevention, U.S. Agency for International Development, U.S. Peace Corps, U.S. Department of Defense, U.S. Office of the Global AIDS Coordinator, the American College of Obstetricians and Gynecologists, Every Mother Counts, Merck for Mothers, the Government of Norway, and the Project C.U.R.E.

The SMGL theory of change, goals, and objectives have been described in detail elsewhere.^15^ SMGL interventions in Uganda and Zambia were accompanied by intensive monitoring and evaluation (M&E) efforts that drew upon the experience of existing global initiatives designed to standardize data-collection methods for monitoring interventions, making decisions, and developing health policies related to maternal and neonatal outcomes and care.^16^ To ensure that M&E efforts were aligned with the country's existing data needs and priorities and that existing data systems were utilized to the greatest extent possible, common guiding principles for M&E, frameworks, and indicators were adopted.^16^ Data systems that documented maternal and neonatal health outcomes accurately and completely were needed to measure changes in key outcomes. Since the necessary data systems to document these outcomes were only partially in place at the outset of SMGL, intensive efforts were made to scale up or establish community- and facility-based data collection systems.

Intensive efforts were made to scale up or establish community- and facility-based data collection to document maternal and neonatal health outcomes.

This article describes the methods employed in Uganda and Zambia to document SMGL results and presents an overview of the changes in intermediate results and health outcomes at the conclusion of the 5-year SMGL initiative. The specific strategies and interventions used in each country to address these goals are described in other articles in this supplement.^15^^,^^17^^–^^19^

## METHODS

### SMGL Implementation Areas

Uganda and Zambia were selected for the SMGL initiative because of their high number of maternal deaths and elevated MMRs, average or below average use of maternal health services (especially around the time of delivery) compared with other countries in the region, government commitments to improving maternal and neonatal survival, and ability to leverage existing U.S. government platforms to promote maternal health and reduce HIV transmission. Before the SMGL initiative, Uganda and Zambia had estimated national MMRs of 438 and 398 maternal deaths per 100,000 live births, respectively.^20^^,^^21^ In Uganda alone, an estimated 4,700 maternal deaths and 35,000 neonatal deaths occurred every year.

Four SMGL-supported districts were designated as 'learning districts' in both Uganda and Zambia. Just prior to implementation of SMGL in 2011, Uganda SMGL-supported districts had a combined population of 1.75 million, with approximately 330,776 women of reproductive age (WRA; women aged 15–49 years) and an estimated 78,000 live births annually (Table 2).^22^ Zambia SMGL-supported districts had a smaller combined population of 925,000, with approximately 194,000 WRA and 37,000 annual live births. Whereas the 4 learning districts in Uganda were contiguous and densely populated, the 4 learning districts in Zambia were geographically dispersed and comprised a much larger, but more sparsely populated, geographic area. At SMGL baseline, Uganda learning districts had more hospitals and high-level health centers (HC IVs) with surgical capacity per capita than the learning districts in Zambia.^23^^,^^24^ Hospitals in SMGL-supported districts in both countries are predominantly government-owned, with a few private, faith-based facilities. Both countries had a regional hospital (Fort Portal Regional Referral Hospital in Uganda and Mansa General Hospital in Zambia) that was part of the SMGL initiative, with catchment areas that extended to neighboring non-SMGL-supported districts. Health centers III in Uganda and all health centers in Zambia were mid-level facilities that provide basic maternity and newborn care and limited emergency obstetric care including some, but not all, of the 7 BEmONC signal functions. Assisted vaginal delivery, in particular, was often not performed in mid-level facilities due to concerns about possible adverse events. Health centers II in Uganda and health posts in Zambia are lower-level primary care facilities that provide antenatal, delivery, and postpartum care and refer complicated births to higher-level facilities.

**TABLE 2.** Baseline SMGL-Supported District Characteristics

Characteristic	Uganda	Zambia
Area (sq. km)	10,851	49,468
Population (2011)^a^	1,750,000	925,198
% of population in rural areas	84%	61%
Number of women of reproductive age (in 2011)^a^	330,776	193,515
Number of expected live births (in 2011)^b^	78,261	37,267
Number of health care facilities, by type (in 2011)		
Health posts	19	16
Health centers without surgical care	72	91
Health centers with surgical care	8	0
District hospitals	7	5
Regional hospital^c^	1	1
Number of facilities, by ownership (in 2011)		
Government	65	106
Private for profit	11	0
Private not-for-profit	31	7
Number of EmONC facilities (in 2011)^d^		
Basic EmONC	3	3
Comprehensive EmONC	7	4

Abbreviations: CEmONC, comprehensive emergency obstetric and newborn care; EmONC, emergency obstetric and newborn care; SMGL, Saving Mothers, Giving Life.

a Based on the 2013 4-district population census in Uganda and the Population and Housing Census 2010 in Zambia projected to 2011 for the 4 SMGL districts.

b In Uganda, expected births were estimated by multiplying the number of women of reproductive age from the 2013 4-district census by age-specific fertility rates from the 2011 Demographic and Health Survey; in Zambia, expected births were derived from the 2010 census crude birth rates.

c Fort Portal Regional Referral Hospital is a 351-bed level-3 referral hospital located in Kabarole district and serving 3 SMGL-supported districts (Kabarole, Kyenjojo, and Kamwenge) and 4 non-SMGL districts (Kasese, Ntoroko, Kyegegwa, and Bundibugyo); Mansa General Hospital is a 352-bed level-2 referral hospital providing care to Luapula province, which, in 2011, included 1 SMGL-supported district (Mansa) and 5 nonsupported districts.

d Facilities were classified based on whether they had, within the previous 3 months, performed the signal functions associated with each level of EmONC care. Because assisted vaginal delivery—using either forceps or vacuum extractor—is relatively uncommon in both Uganda and Zambia, some facilities were classified as fully providing EmONC care even if they did not perform assisted vaginal deliveries within the past 3 months (EmONC-1). In Uganda, district and regional hospitals and health centers with surgical capacity (health centers IV) are designated as CEmONC facilities, able to perform each of the 9 signal functions and serving about 100,000 population; in Zambia, only district and higher-level hospitals are designated to provide CEmONC care.

The SMGL initiative was implemented in phases: Phase 0 (pre-implementation planning in 2011–2012), Phase 1 (June 2012 to December 2013), and Phase 2 (January 2014 to October 2017). Phase 1 consisted of rapidly scaled-up facility and community interventions (“the big push”) to address the 3 delays.^15^ Phase 2 aimed to continue and consolidate successful interventions introduced in Phase 1; improve quality of care, including care for sick and small newborns; and further refine M&E methods and surveillance activities in the learning districts. Additional districts in both countries adopted the SMGL model, except for the M&E approaches.^15^ To evaluate SMGL's impact, comparisons of maternal and perinatal outcomes in the learning districts were made between the 12-month baseline period (June 2011 to May 2012) prior to SMGL implementation and the endline period (January to December 2016).

The baseline and endline evaluations used similar M&E approaches to measure progress and outcomes: health facility assessments (HFAs), facility pregnancy outcome monitoring with enhanced identification of maternal deaths, and, at the SMGL-supported district population level, community-based maternal death identification with Reproductive Age Mortality Studies (RAMOS) in Uganda and censuses in Zambia, which included verbal autopsies for suspected maternal deaths (Table 3). Each of the M&E data collection and analytic approaches is described in greater detail below.

**TABLE 3.** SMGL Indicator Baseline and Endline Data Sources in Uganda and Zambia SMGL-Supported Districts

Period and Indicator	Uganda			Zambia	
	Community	Health Center IV and Hospitals	Health Centers III and II	Community	Health Centers and Hospitals
Baseline (June 2011–May 2012)					
Routine and emergency obstetric care indicators	--	HFA	HFA	--	HFA
Institutional deliveries (vaginal and cesarean deliveries)	--	Individual outcome data and triangulation of facility registers (POMS)	Facility aggregate outcome data	--	HFA and facility aggregate outcome data
Direct obstetric complications prevalence rates	--	POMS and RAPID	Facility aggregate outcome data	--	HFA and facility aggregate outcome data
Stillbirth and predischarge neonatal mortality rates	--	POMS and RAPID	Facility aggregate outcome data	--	HFA and facility aggregate outcome data
Cause-specific maternal mortality and case fatality rates	--	POMS and RAPID triangulated with RAMOS	Facility aggregate outcome data	--	Facility aggregate outcome data triangulated with census-identified maternal deaths
Population maternal mortality ratios	RAMOS	--	--	4-district census^a^	--
Endline (January–December 2016)					
Routine and emergency obstetric care indicators	--	HFA	HFA	--	HFA
Institutional deliveries (vaginal and cesarean deliveries)	--	POMS	POMS	--	HFA and facility aggregate outcome data
Direct obstetric complications prevalence rates	--	POMS and RAPID	POMS and RAPID	--	HFA and facility aggregate outcome data
Stillbirth and predischarge neonatal mortality rates	--	POMS and RAPID	POMS and RAPID	--	HFA and facility aggregate outcome data
Cause-specific maternal mortality and case fatality rates in facilities	--	POMS and RAPID triangulated with RAMOS	POMS and RAPID triangulated with RAMOS	--	Facility MDSR; individual cases triangulated with census-identified maternal deaths
Population maternal mortality ratios	RAMOS	--	--	4-district census^a^	--

Abbreviations: HFA, health facility assessment; MDSR, maternal death surveillance and response; POMS, Pregnancy Outcome Monitoring System; RAPID, Rapid Ascertainment Process for Institutional Deaths; RAMOS, Reproductive Age Mortality Studies.

a Conducted in 2012 and 2017 for the previous 18 months; 12-month pre-census population maternal mortality ratios were estimated after adjustments for underreporting of population and births.

Each country's baseline and endline evaluations used similar M&E approaches to measure progress and outcomes.

### Health Facility Assessments

At SMGL baseline and endline, each country conducted health facility assessments (HFAs) in all facilities that provided childbirth care in the SMGL-supported districts, using a modified version of the standard EmONC HFA questionnaire originally developed by the Averting Maternal Death and Disability program at Columbia University.^25^ The HFAs gathered data on maternity care infrastructure, human resources, and adherence to safe motherhood protocols and practices, drugs, equipment, and supplies. The HFAs also characterized facility EmONC status—defined as performance of 7 BEmONC or 9 CEmONC signal functions in the 3 months prior to the HFAs—and assessed capacity and use of transport for emergency referrals. The number of health facilities performing deliveries varied in each country over the 5-year initiative. The HFA results presented here were compiled only from those facilities that maintained delivery capacity from baseline to endline (105 in Uganda and 110 in Zambia).

The HFA baseline results were used to document baseline status and identify programmatic needs for SMGL. The results also informed the distribution of human and financial resources to strengthen infrastructure and other facility capacities, particularly during Phase 1 of the initiative.^26^ The results of the endline HFAs, conducted in November 2016, were used to assess changes in infrastructure and capacity at the end of the initiative and to guide planning for post-SMGL sustainability. Baseline and endline indicators of changes in health care facility infrastructure, availability of medications and supplies, and EmONC functions and labor management were calculated as the percentages of all facilities that reported positive responses on the HFA indicators, with the exception of indicators that are reported as complete enumerations.

HFA results documented baseline status and program needs and informed the distribution of human and financial resources to strengthen infrastructure and other facility capacities.

### Facility Pregnancy Outcome Monitoring

Individual and aggregated retrospective pregnancy outcome data, including identification of maternal deaths in facilities, were collected periodically by trained health facility staff and SMGL M&E personnel in both countries using enhanced data collection tools. In 2011, Uganda and Zambia had just started to use an electronic aggregated health service data platform (District Health Information System, version 2), which did not cover all health facilities. To address this, SMGL M&E teams developed standard abstraction forms and operation procedures for ongoing data collection of health service and outcome indicators. SMGL-supported facility monitoring led to improvements in tracking routine service delivery indicators as part of the newly established district data platform.

In Uganda, SMGL-supported facilities that provided CEmONC implemented individual-level Pregnancy Outcome Monitoring Surveillance (POMS) data collection on maternal and newborn outcomes, including information on obstetric surgeries. As part of POMS, a package of standard tools was developed and used to obtain comprehensive maternal and reproductive health information: (1) electronic abstraction of all individual pregnancy outcomes found in labor and delivery registers, (2) abstraction forms to triangulate data on complications and obstetric surgeries from multiple sources, and (3) standard operation procedures to perform data abstraction and data entry. Because SMGL-supported facilities used ward-specific log books rather than centralized health records, POMS data from hospitals and HC IVs were triangulated with patient logs from various sources—such as labor and delivery, postpartum, female ward, surgical, admission/discharge registers, and hospital morgues—within each health facility.^27^ Trained SMGL M&E and clinical staff collected information on maternal characteristics, type of delivery, pregnancy outcomes, and up to 3 maternal complications at the time of each delivery. The most immediately life-threatening complication was used to analyze maternal morbidities and calculate case fatality rates (CFRs) from direct obstetric causes. Although data on early pregnancy outcomes—spontaneous and induced abortions and ectopic pregnancies—were also individually collected, they were not included in the calculation of the severe direct obstetric complications and CFRs unless they led to maternal demise. This approach was used to ensure that only severely complicated early pregnancy outcomes are examined and yield conservative estimates of met need for obstetric complications and CFRs.

In Uganda, SMGL-supported facilities triangulated POMS data and patient logs within each facility.

In lower-level delivery facilities in Uganda, aggregated outcome data from maternity registers were collected at baseline. By Phase 2 of SMGL, the individual-level POMS approach was expanded to all delivery facilities and individual delivery and pregnancy loss data were collected every 3 months using a Microsoft Access-based electronic data management system. Starting in 2013, the Ugandan Ministry of Health, in collaboration with the implementing partners, introduced an ongoing maternal death surveillance and response (MDSR) system in SMGL-supported health facilities and communities, with the goal of more accurately identifying and ascertaining maternal deaths.

In Uganda, detection of facility maternal deaths was enhanced using the Rapid Ascertainment Process for Institutional Deaths (RAPID) methodology,^28^ in which all health facility records related to deaths among WRA were reviewed. RAPID data collection was conducted periodically in hospitals and HC IVs by Ugandan and U.S. Centers for Disease Control and Prevention obstetricians, and collected data were cross-checked with POMS data. While conducted separately, RAPID enhanced the capacity of facility-based MDSR to identify and review additional facility maternal deaths.

In Zambia, aggregate facility maternal and perinatal outcome data were collected at baseline by SMGL M&E teams from each implementing partner in conjunction with the baseline HFA data collection. After SMGL interventions were introduced, monthly collection of aggregated facility outcomes data continued through the end of Phase 1 (December 2013). Facility data abstraction forms were used to compile aggregated data primarily from maternity registers. However, data abstraction forms and the completeness of case detection varied among implementing partners. In Phase 2, the periodicity of data abstraction changed from monthly to quarterly and a unified electronic data abstraction tool was implemented.

In Zambia, facility data abstraction forms were used to compile aggregated data primarily from maternity registers.

Starting in mid-2015, enhanced case detection and an audit of each maternal death became mandatory in all Zambian health facilities as part of newly implemented national maternal mortality surveillance. SMGL monitoring included audited maternal deaths in the estimation of endline facility maternal mortality.

In both countries, and in accordance with the global MDSR guidance,^29^ baseline and endline measurements of maternal deaths in SMGL-supported facilities were derived by cross-checking multiple facility and community data sources (as further described) in order to capture a complete list of maternal deaths. Maternal deaths captured in these sources include those due to direct and indirect obstetric causes. Direct, indirect, and cause-specific MMRs in facilities were calculated as the number of cause-specific maternal deaths per 100,000 live births. WHO guidelines for using the 10th revision of the International Classification of Diseases to the classification of maternal mortality (ICD-MM) were applied to determine the underlying cause of death.^30^ However, this was complicated by the inclusion of the deaths of pregnant or postpartum women who lived outside SMGL-supported districts—and, thus, were not exposed to the SMGL interventions—in the count of maternal deaths in the SMGL-supported facilities.

Facility-based pregnancy outcome data were used to estimate other standard indicators of monitoring emergency obstetric care, such as the cesarean delivery rate, met need for emergency obstetric care, the direct obstetric CFR, and the facility MMR.^5^^,^^10^ The cesarean delivery rate was defined as the proportion of deliveries by cesarean delivery of total district births. Met need for emergency obstetric care in all facilities was defined as the proportion of all women expected to have developed severe obstetric complications (estimated at 15%)^10^ who were treated in any health facility, and the met need for emergency obstetric care in EmONC facilities was represented by the proportion of expected severe obstetric complications that were treated in a fully functioning EmONC facility. The direct obstetric CFR was defined as the proportion of all women admitted to all facilities and to EmONC facilities with a given severe complication who died before discharge. The facility MMR was calculated as the number of maternal deaths per 100,000 live births in SMGL-supported facilities.

Throughout the SMGL initiative, the number of stillbirths and predischarge neonatal deaths among babies weighing ≥1000 grams were monitored from information recorded in facility maternity registers. In contrast to the monitoring of maternal deaths for SMGL, identification of perinatal deaths was not enhanced through triangulation of multiple data sources, audits were less widespread, and underlying medical and nonmedical causes were not consistently available. Individual-level data on maternal and delivery characteristics, Apgar scores, and birthweight were available only in Uganda; similar data were collected as aggregate counts in Zambia. Although facility HFAs reported whether they had performed neonatal resuscitation, information about successful neonatal resuscitation was not available for either country.

The facility perinatal mortality rate was calculated as the number of stillbirths and predischarge neonatal deaths among births delivered in facilities divided by the total number of births (live births and stillbirths) in SMGL-supported facilities. Similarly, the total facility stillbirth rate (SBR) was calculated as the total number of facility stillbirths per 1,000 facility births. In Uganda, where timing of fetal death was captured, it was possible to calculate the intrapartum SBR as the number of intrapartum stillbirths (those occurring after the onset of labor but before birth) divided by the total number of births per 1,000 births. Finally, the predischarge neonatal mortality rate (NMR) was calculated as the number of facility neonatal deaths divided by the total number of facility live births per 1,000 live births.

### Community-Based Maternal Death Identification

In Uganda, retrospective RAMOS were conducted in SMGL-supported districts to capture community-level maternal deaths at baseline, end of Phase 1, and endline. At baseline, trained village health teams used community registers to identify and compile lists of WRA deaths in the prior 18-month period. Deaths were investigated using a 1-page screening tool to identify WRA who had been pregnant during the 2 months preceding death. Caretakers of women who died while pregnant or postpartum were interviewed using a standardized verbal autopsy protocol, which explores circumstances and potential causes of maternal death.^29^^,^^31^ At the end of Phase 2, the trained interviewers used an expanded RAMOS questionnaire that collected data on household composition, lifetime and recent pregnancy events among all WRA residing in the household, and all deaths in the household since January 2016. Households that reported WRA deaths were further asked to identify if deaths occurred during pregnancy, delivery, or postpartum using the baseline 1-page screening tool. Verbal autopsy teams conducted interviews with caregivers to women whose deaths were associated with pregnancy. At both baseline and endline, verbal autopsy data were analyzed independently by 2 physicians trained to assign underlying cause of death, with a third physician opinion sought when no consensus on cause of death could be reached. They then issued a consensus standard WHO death certificate for each verbal autopsy. Only maternal deaths that occurred during the baseline and endline periods were included in the analyses.

In Uganda, retrospective RAMOS were conducted in SMGL-supported districts to capture community-level maternal deaths at baseline, end of Phase 1, and endline.

In Zambia, community-level maternal mortality data were collected using household population censuses conducted in 2012 and 2017. The primary aim of the censuses was to assess the baseline mortality for WRA—including maternal mortality—and the change between the 2 time points. To enable calculation of maternal mortality rates and ratios, the household census data provided the number of WRA, the number of WRA deaths, and the number of live births in the population within the 12-month period before each census. For the 2012 census, the recent period used for WRA deaths and births was March 2011–February 2012; for the 2017 census, the recent period was July 2016–June 2017. A series of questions was asked about each person who was a usual member of the household and had died recently (since October 1, 2010, for the 2012 census and since January 1, 2016, for the 2017 census), including the age, sex, and dates of birth and of death. For each death of a woman aged 12 to 49 years, additional questions were asked about whether the woman had died when pregnant, during childbirth, or within 2 months after the end of a pregnancy.

For each death of women aged 12 to 49 years, a verbal autopsy interview was conducted with a member of the household to record information about the circumstances, signs, and symptoms experienced by the deceased before she died. Teams of trained physicians reviewed the verbal autopsy interview responses and coded them to assign causes of death within both the baseline and endline censuses. To compensate for underreporting of deaths in reported numbers of all deaths of women aged 15 to 49 years and on maternal deaths from the baseline and endline censuses, standard adjustments to the data were made.

We compared census-based measurements of population, births, and deaths in the 4 SMGL-supported districts with external sources and assessed that they were incomplete, particularly at baseline.^20^^,^^21^^,^^32^^–^^34^ We adjusted the 4 district mortality completeness using the General Growth Balance method.^35^ The adjustment factors were derived from fitting a line to a series of observed and predicted mortality rates for different age groups using the most recent national censuses and the United Nations Census Pregnancy-Related Mortality (CensusPRM) workbook for estimating maternal mortality from census data.^36^

The proportion of deaths among women of reproductive age that are due to maternal causes was estimated using the verbal autopsy data and applied to adjusted numbers of deaths to WRA to obtain the estimated number of maternal deaths. Likewise, the proportions of maternal deaths due to specific causes were applied to the estimated number of all maternal deaths in a reporting period to estimate the number of maternal deaths by cause.

In order to establish a more comprehensive count of maternal deaths in facilities, community maternal death data in both countries were cross-checked with deaths reported through facility monitoring. A probabilistic match between information from verbal autopsies and from facility monitoring using place, cause, and month of death was completed. If a facility death was reported in a verbal autopsy but was not matched to a death recorded in the facility's monthly monitoring statistics, the death was classified as an additional facility-based death and added to the facility count of maternal deaths.

Population-based MMRs were computed using information collected through verbal autopsies in Uganda and Zambia at baseline and endline. Total and cause-specific MMRs were calculated after classifying causes of maternal death in accordance with ICD-MM.^29^ Zambia baseline verbal autopsy data were reclassified at the endline using ICD-MM, which was initially only used at endline. This resulted in an increase in the counts of Zambia maternal deaths identified in the baseline census and a corresponding increase in the baseline facility-based maternal mortality previously published.^16^ Direct, indirect, and cause-specific population MMRs were calculated as the number of cause-specific deaths in the SMGL-supported districts per 100,000 live births to WRA in these districts. The annual rate of SMGL MMR reduction (ARR) was calculated as: ARR=log(MMR_endline_/MMR_baseline_)/5*100. This is consistent with WHO methodology to estimate MMR ARRs both globally and at the country level.^1^

Population-based MMRs were computed using information collected through verbal autopsies in Uganda and Zambia at baseline and endline.

Response rates for verbal autopsies were very high in both countries. In Uganda, only 6 suspected maternal deaths identified in the baseline RAMOS and 2 deaths in the endline were not followed by an interview due to household dissolution or relocation. There were no refusals to participate in the baseline and endline RAMOS studies. In Zambia, several suspected maternal deaths were not followed by verbal autopsies (11 at baseline and 18 at endline). Refusals were encountered from 2 and 5 households, respectively. However, population maternal mortality data from Zambia are adjusted estimates based on the application of the General Growth Balance method to compensate for underreporting of WRA deaths and the estimated proportion of deaths among WRA that are due to maternal causes to derive maternal deaths.

### Population Denominators

Calculation of population MMRs and selected EmONC indicators requires external population data. District-wide censuses in Zambia (2012 and 2017) and Uganda (2013 and 2017) were conducted by SMGL to enumerate households, population, and WRA. Enumerations were projected back to estimate the 2011 population using the inverse growth coefficient derived from the intercensal population growth rate provided by the countries' national statistics bureaus.^32^^,^^33^ The baseline number of live births in Zambia districts was estimated by applying crude birth rates to the baseline district populations—directly derived from the 2010 national census. The endline live births were estimated by applying district-specific facility delivery rates calculated from the 2017 SMGL census to the endline district population. In Uganda, the number of live births was estimated by applying age-specific fertility rates among WRA enumerated in 2013 and 2017 in Uganda districts. For both countries, we calculated MMRs in facilities using the number of live births in facilities as the denominator and population-based MMRs using the estimated number of live births in the SMGL-supported districts.

District-wide censuses in Zambia and Uganda were conducted by SMGL to enumerate households, population, and WRA to facilitate calculation of population MMRs.

### Statistical Analyses

The results shown here were based on 4 district data analyses performed for each country. They were based on the total population and total number of health facilities in the SMGL-supported districts in each country. They were not a sample and are not representative of a larger population in the country. The pregnancy outcomes in facilities, including institutional mortality rates and ratios, were based on complete enumeration of deaths identified in facilities, so they were not subject to sampling error. However, the rates and ratios may be affected by random variation and changes in case detection.^37^ The following statistical tests were used when testing the difference between the Phase 0 and Phase 2 results. For the mortality rates and ratios, the error was modeled assuming deaths and births to be distributed according to a Poisson distribution. A *z* statistic, *z*=_√_SE(*MMR_baseline_*)^2^ + SE(*MMR_endline_*),^2^ was used to calculate the *P* value of the difference between the baseline and endline MMRs, both in facilities and when comparing population MMRs.^38^ Similarly, changes in other core indicators, based on complete counts of events during the 2 periods, were also estimated using *z* statistics for significance testing. Finally, for the indicators that capture facility functionality, infrastructure, and availability of supplies, the McNemar's test, which is appropriate for dichotomous responses for matched pairs of data collected at different time points, was used to test for significant differences.^39^ Results were considered significant if *P*<.05.

### Ethical Considerations

The study protocol was approved by, and complied with, Uganda and Zambia Ministries of Health procedures for protecting human rights in research, and was deemed nonresearch by the U.S. Centers for Disease Control and Prevention Human Research Protection Office of the Center for Global Health. Written informed consent was obtained for respondents in all households and among women for the census and RAMOS interviews. For the verbal autopsies, written consent among the caregivers of the deceased subjects was obtained after informing the caregivers about the purpose and public health importance of the research, selection procedures, voluntary participation, and confidentiality. Interviews were scheduled no sooner than 6 weeks after the death occurred.

## RESULTS

Each country's SMGL-supported districts achieved improvements in numerous aspects of facility infrastructure and provision of delivery care (Table 4). By the end of the initiative, the proportion of delivery facilities that provided delivery care 24 hours a day/7 days a week had increased significantly by 41% in Zambia (from 68.2% to 96.4%) but had not increased significantly in Uganda, where the baseline 80% of facilities providing care 24 hours a day/7 days a week was already comparatively high. The proportion of facilities with uninterrupted electricity increased significantly in both countries, from 57.1% to 96.2% in Uganda and from 55.5% to 96.2% in Zambia. Virtually all SMGL-supported facilities in both countries had running water and functional communications systems by the end of the initiative. In Zambia, where distances to facilities were greatest, transport capacity at the facility level increased by 31%, from 55.5% to 72.7%. Facility obstetric capacity—defined as having a sufficient number of beds so obstetric patients do not share beds—increased significantly in Uganda (from 35.2% to 91.4%) but remained insufficient at endline in Zambia, where about one-quarter of facilities reported that obstetric patients have to share beds.

**TABLE 4.** Selected Facility Characteristics and Interventions at Baseline and Endline in Uganda and Zambia SMGL-Supported Districts

Facility Characteristic/Intervention	Uganda (n=105 facilities)				Zambia (n=110 facilities)			
	Baseline^a^^,^^b^	Endline^a^^,^^b^	% Change^c^	Sig. Level^d^	Baseline^a^^,^^b^	Endline^a^^,^^b^	% Change^c^	Sig. Level^d^
**Facility infrastructure**								
Availability of delivery services 24 hours a day/7 days a week	80.0	87.6	+10	NS	68.2	96.4	+41	***
Uninterrupted electricity available	57.1	96.2	+69	***	55.5	92.7	+67	***
Running water available	76.2	100.0	+31	N/A	90.0	97.3	+8	**
Functional communications available^e^	93.3	99.0	+6	**	44.6	100.0	+124	N/A
Transportation available^f^	61.0	59.0	−3	NS	55.5	72.7	+31	***
Sufficient number of obstetric beds	35.2	91.4	+160	***	62.7	73.6	+17	NS
Women do not deliver on the floor	85.7	91.4	+7	NS	71.3	83.8	+18	NS
Mother shelter present	0	3.9	NA	N/A	28.8	48.8	+69	***
**Availability of medications and supplies**								
No stock-out in last 12 months: magnesium sulfate^g^	47.6	63.8	+34	***	20.0	43.0	+115	***
No stock-out in last 12 months: oxytocin^g^	56.2	81.9	+46	***	75.3	75.0	−0.4	NS
HIV rapid test kits currently available^g^^,^^h^	70.5	79.0	+12	NS	82.5	93.8	+14	**
At least 1 long-acting reversible family planning method currently available	41.0	55.2	+35	***	20.0	71.3	+257	***
**EmONC functions and labor management**								
Number of functioning CEmONC facilities	7	17	+143	N/A	4	5	+25	N/A
Number of functioning BEmONC facilities	3	9	+200	N/A	3	8	+167	N/A
Number of facilities with partial BEmONC^i^	19	34	+79	N/A	22	29	+32	N/A
Use of partograph to monitor labor	33.3	92.4	+178	***	NA	92.7	NA	NA
Active management of third stage of labor	75.2	96.2	+28	***	71.8	95.5	+33	***
Use of parenteral antibiotics in last 3 months	85.7	92.4	+8	NS	79.1	73.6	−7	NS
Use of parenteral oxytocin in last 3 months	69.5	98.1	+41	***	90.9	95.5	+5	NS
Use of parenteral anticonvulsants in last 3 months	48.6	34.3	−29	**	44.6	40.0	−10	NS
Perform newborn resuscitation in last 3 months	34.3	87.6	+155	***	27.3	74.6	+173	***
Perform manual removal of placenta in last 3 months	28.6	54.3	+90	***	39.1	30.0	-23	NS
Remove retained products in last 3 months	19.0	61.9	+226	***	17.3	49.1	+184	***
Perform assisted vaginal delivery in last 3 months	4.8	10.5	+119	NS	10.0	15.5	+55	NS
Perform surgery (cesarean delivery) (HC IV or higher) in last 3 months	7.6	16.2	+113	***	3.6	4.6	+28	NS
Perform blood transfusion (HC IV or higher) in last 3 months	8.6	16.2	+88	***	5.5	4.6	−16	NS
Perform maternal death reviews^i^	6.7	32.4	+384	***	42.5	75.0	+76	**
**Health facility has associated community volunteers**	18.3	91.5	+400	***	63.8	96.3	+51	***

Abbreviations: BEmONC, basic emergency obstetric and newborn care; CEmONC, comprehensive emergency obstetric and newborn care; EmONC, emergency obstetric and newborn care; HC, health center; Sig., significance.

Note: All data reported as percentages unless otherwise noted.

a Baseline period is June 2011–May 2012; endline period is January–December 2016.

b Baseline and endline results are percentages of all facilities, unless otherwise noted.

c Percent change calculations based on unrounded numbers.

d Asterisks indicate significance level of the difference between baseline and endline outcomes using McNemar's exact test, as follows: ***P<.01, **P<.05, NS = not significant. NA = data not available. N/A = not applicable.

e Uganda: facility-owned landline, cell, two-way radio, or individual had cell phone; Zambia: two-way radio, landline, or cell phone with service.

f Uganda: available and functional motorized vehicle with fuel today and funds generally available; Zambia: motor vehicle, motorcycle, or bicycle.

g Zambia: Kalomo facilities did not collect the information and were excluded from the analysis.

h Uganda: Rapid HIV test was used in maternity ward in the last 3 months (does not indicate current availability).

i Percentage of health centers that performed 4 to 5 basic emergency obstetric care interventions in the past 3 months.

Virtually all SMGL-supported facilities in both countries had running water and functional communications systems by the end of the initiative.

Availability of lifesaving medications and supplies improved, with significant increases in the percent of facilities in both countries reporting no stock-outs of magnesium sulfate (Table 4). Despite the significant increase, however, at endline only 63.8% of facilities in Uganda and 43.0% in Zambia reported no magnesium sulfate stock-outs. The availability of oxytocin also improved in Uganda, where the percent of facilities reporting no stock-outs increased significantly from 56.2% to 81.9%. The percent of facilities having no stock-outs of oxytocin in Zambia, however, did not change significantly. Because the availability of HIV rapid test kits in facilities was already over 70% in Uganda and over 83% in Zambia at SMGL baseline, no significant increase of their availability was reported in either country. However, the availability of long-acting reversible family planning methods did increase significantly in both countries over the course of SMGL.

Both countries' SMGL-supported districts documented increases in the number of health care facilities that reported having provided CEmONC and BEmONC signal functions in the 3 months prior to the baseline and endline HFAs. Additionally, a larger number of non-EmONC facilities were able to perform 4 to 5 of the 7 basic EmONC signal functions. At endline, more than 92% of facilities in both countries reported routine partograph use to monitor labor, with partograph use almost tripling in Uganda (from 33.3% to 92.4%, a significant increase). The practice of active management of the third stage of labor also increased significantly and was nearly universal in both counties at endline (96.2% in Uganda and 95.5% in Zambia). Use of parenteral antibiotics did not increase significantly in either country; however, use of parenteral oxytocin increased significantly in Uganda but not in Zambia. Use of parenteral magnesium sulfate remained low in both countries, with use declining slightly but significantly in Uganda. In both countries, SMGL-supported facilities reported significant increases in performance of neonatal resuscitation—from 34.3% to 87.6% in Uganda and from 27.3% to 74.6% in Zambia. In Uganda, significant increases were seen in the percentage of facilities that reported having performed manual removal of the placenta, removal of retained products, cesarean deliveries, and blood transfusions. In Zambia, significant increases were only found in performance of removal of retained products. The proportion of health facilities conducting maternal death reviews, as mandated by government health policies in both countries since 2009, increased significantly from 6.7% to 32.4% (from 7 to 34 facilities) in Uganda and from 42.5% to 75.0% (from 47 to 82 facilities) in Zambia.

In both countries, SMGL-supported facilities reported significant increases in performance of neonatal resuscitation.

SMGL-supported facilities in both countries documented significant improvement over the course of the SMGL initiative in numerous health outcome-related indicators (Table 5). For example, the volume of facility deliveries increased by 71% in Uganda and by 74% in Zambia and facility delivery rates increased significantly, with a 47% increase in facility deliveries in Uganda (from 45.5% to 66.8% of district births) and a 44% increase in Zambia (from 62.6% to 90.2% of district births). Delivery rates in EmONC facilities also increased significantly, by 45% in Uganda and 12% in Zambia.

**TABLE 5.** Maternal Health Outcomes in Facilities at Baseline and Endline in Uganda and Zambia SMGL-Supported Districts

Maternal Health Outcomes	Uganda				Zambia			
	Baseline	Endline	% Change	Sig. Level^a^	Baseline	Endline	% Change	Sig.Level^a^
Number of live births – all facilities	33,492	57,355	+71	N/A	21,914	38,174	+74	N/A
Institutional delivery rate – all facilities (%)	45.5	66.8	+47	***	62.6	90.2	+44	***
Institutional delivery rate – EmONC facilities (%)	28.2	41.0	+45	***	26.0	29.1	+12	***
Number of obstetric complications treated^b^	5,256	8,458	+61	N/A	1,844	1,979	+7	N/A
Cesarean delivery rate as a proportion of all births (%)	5.3	9.0	+71	***	2.7	4.8	+79	***
Met need for emergency obstetric care – all facilities (%)	46.3	64.7	+40	***	34.1	30.6	−10	***
Met need for emergency obstetric care – EmONC facilities (%)	39.2	62.1	+58	***	25.8	23.1	−11	***
Direct obstetric case fatality rate – all facilities (%)	2.6	1.7	−37	***	3.7	3.2	−12	NS
Direct obstetric case fatality rate – EmONC facilities (%)	2.9	1.6	−45	***	2.9	3.8	+31	NS
Facility MMR, overall (per 100,000 live births)	534	300	−44	***	370	231	−38	***
Direct obstetric causes MMR	415	244	−41	***	310	168	−46	***
Obstetric hemorrhage MMR^c^	131	77	−42	***	119	42	−65	***
Puerperal infection/sepsis MMR^d^	75	47	−37	NS	NA	NA	N/A	N/A
Obstructed labor MMR^e^	72	56	−22	NS	59	31	−47	NS
Abortion-related MMR^f^	63	23	−64	***	NA	NA	N/A	N/A
Preeclampsia/eclampsia MMR	45	26	−42	NS	NA	NA	N/A	N/A
Other major direct obstetric causes MMR^g^	30	16	−47	NS	132	94	−29	NS
Indirect obstetric causes MMR^h^	119	56	−53	***	59	63	+6	NS
Facility perinatal mortality (per 1,000 births)	39.3	34.4	−13	***	37.9	28.2	−26	***
Total stillbirth rate (per 1,000 births)	31.2	27.0	−13	***	30.5	19.6	−36	***
Intrapartum stillbirth rate (per 1,000 births)	22.4	14.3	−36	***	NA	NA	N/A	N/A
Predischarge neonatal mortality rate (per 1,000 live births)	8.4	7.6	−10	NS	7.7	8.7	+14	NS

Abbreviations: EmONC, emergency obstetric and newborn care; MMR, maternal mortality ratio; Sig., signficance.

a Asterisks indicate significance level of the difference between baseline and endline outcomes for all facilities combined, using a *z* statistic to calculate the *P* value of the difference, as follows: ***P<.01, **P<.05, NS = not significant. NA = data not available. N/A = not applicable.

b Excludes early pregnancy complications (e.g., abortion-related complications and ectopic pregnancy).

c Includes antepartum, intrapartum, and postpartum hemorrhage.

d Infection of the genital tract occurring at any time between the onset of the rupture of membranes or labor and the day of death in facility; in Zambia, these maternal deaths were classified as deaths due to “other major direct complication.”

e Obstructed and prolonged labor including rupture of the uterus.

f Deaths after induced and spontaneous abortions.

g In Uganda, it includes deaths due to embolism, anesthesia, and ruptured ectopic pregnancy; in Zambia, it includes these conditions plus deaths due to puerperal infections, eclampsia/preeclampsia, and abortion.

h Includes HIV-, TB-, and malaria-related maternal deaths and those due to other medical conditions aggravated by pregnancy or postpartum.

The number of major direct obstetric complications treated in facilities also increased in both countries, including reported facility cases of obstetric hemorrhage, prolonged or obstructed labor, ruptured uterus, sepsis, preeclampsia/eclampsia, and other severe direct obstetric complications (data not shown). The met need for EmONC in all facilities—meaning the proportion of all women with major direct obstetric complications in the population treated in health facilities—increased by 40% in Uganda (from 46.3% to 64.7%) but declined by 10% in Zambia (from 34.1% to 30.6%). Similarly, the met need in EmONC facilities—meaning the proportion of all women with major direct obstetric complications in the population treated in EmONC facilities—increased by 58% in Uganda but declined slightly in Zambia. Cesarean delivery rates in the SMGL-supported districts increased by 71% in Uganda (from 5% to 9%) and 79% in Zambia (from 3% to 5%).

Facility MMRs declined significantly in both countries, from 534 to 300 maternal deaths per 100,000 live births in Uganda facilities (a 44% decline) and from 370 to 231 per 100,000 live births in Zambia (a 38% decline) (Table 5). In all, facility maternal mortality due to direct obstetric causes declined by 41% in Uganda and 46% in Zambia. The facility MMR for obstetric hemorrhage decreased from 131 to 77 maternal deaths per 100,000 in Uganda (a 42% decline) and from 119 to 42 maternal deaths per 100,000 in Zambia (a 65% decline). In addition, maternal mortality due to postabortion complications fell significantly in Uganda facilities from 63 to 23 maternal deaths per 100,000 (a 64% decline). Although the direct obstetric CFR in all facilities declined from 2.6% to 1.7% in Uganda (a significant 37% decline), it did not change significantly in Zambia. At endline, neither country's direct obstetric CFR reached the 1% upper limit established by WHO^10^; Zambia's direct obstetric CFR remained especially high at 3.2%.

In Uganda, the facility perinatal mortality rate declined significantly from 39.3 to 34.4 perinatal deaths per 1,000 births, a 13% decline. The total SBR in Uganda declined by 13%, from 31.2 to 27.0 stillbirths per 1,000 births, due to reduction in the intrapartum SBR, which declined by 36% (from 22.4 to 14.3 intrapartum stillbirths per 1,000 births); antepartum SBR increased from 8.8 to 12.7 antepartum stillbirths per 1,000 births. In Zambia, the facility perinatal mortality rate declined significantly from 37.9 to 28.2 perinatal deaths per 1,000 births, a 26% decline, and the total SBR declined from 30.5 to 19.6 stillbirths per 1,000 live births. Neither country achieved a significant decline in the predischarge NMR, with final rates of 7.6 neonatal deaths per 1,000 live births in Uganda and 8.7 neonatal deaths per 1,000 live births in Zambia.

At the district population level, the total number of maternal deaths in Uganda dropped from 342 at baseline to 222 at endline (Table 6). The associated Uganda SMGL population-based MMR declined significantly—from 452 to 255 maternal deaths per 100,000 live births, a reduction of 44% (Table 6). This corresponds with an ARR of 11.5% per year. In the Zambia SMGL-supported districts, the adjusted number of maternal deaths decreased from 200 to 135 maternal deaths, corresponding to a reduction in the MMR from 480 to 284 deaths per 100,000 live births, a 41% decline, and an ARR of 10.5%. In SMGL-supported districts, the reduction in maternal mortality was largely driven by declines in direct obstetric causes, with population-level direct obstetric MMRs declining significantly in Uganda (49%) and Zambia (40%). Significant declines in cause-specific mortality were observed in Uganda for obstetric hemorrhage (a 45% decline), obstructed labor (a 36% decline), eclampsia (a 51% decline), postabortion complications (a 67% decline), and other direct causes (a 67% decline). In Zambia, district maternal deaths due to obstetric hemorrhage declined significantly (a 66% decline) as did deaths due to obstructed labor (an 87% decline). Documented changes in indirect obstetric MMRs were not significant in either country. Maternal mortality fell significantly during the intrapartum period and up to 24 hours postpartum in both countries (by 72% in Uganda and 46% in Zambia). Declines in antepartum mortality—before the onset of labor—and greater than 24 hours postpartum were not statistically significant.

**TABLE 6.** Changes in District-Wide Numbers of Maternal Deaths and Maternal Mortality Ratios (per 100,000 Live Births) in Uganda and Zambia SMGL-Supported Districts, by Cause and Timing of Death

	Uganda				Zambia			
	Baseline	Endline	% Change	Sig. Level^a^	Baseline	Endline	% Change	Sig. Level^a^
Number of maternal deaths	342	222	N/A	N/A	200	135	N/A	N/A
Total MMR^b^^,^^c^	452	255	−44	***	480	284	−41	***
Cause of death (MMRs)^d^								
Direct obstetric causes	382	195	−49	***	364	220	−40	***
Obstetric hemorrhage	128	70	−45	***	131	45	−66	***
Obstructed labor (including uterine rupture)	71	46	−36	**	44	6	−87	**
Preeclampsia/eclampsia	58	29	−51	***	36	22	−39	NS
Puerperal infection/sepsis	33	21	−37	NS	29	42	+44	NS
Abortion-related	42	14	−67	***	66	64	−2	NS
Other direct obstetric causes	49	16	−67	***	58	42	−28	NS
Indirect obstetric causes	70	60	−15	NS	116	64	−45	NS
Timing of death (MMRs)^e^								
Antepartum	66	53	−20	NS	109	59	−46	NS
Intrapartum and immediate postpartum (up to 24 hours)	224	62	−72	***	196	106	−46	**
>24 hours–42 days postpartum	161	140	−13	NS	175	120	−31	NS

Abbreviations: MMR, maternal mortality ratio; P/F, parity/fertility; Sig., significance.

a Asterisks indicate significance level of the difference between baseline and 2016 MMRs, using a *z* statistic to calculate the *P* value of the difference, as follows: ***P<.01, **P<.05, NS = not significant. N/A = not applicable.

b Uganda MMRs are direct estimates for the baseline (June 2011–May 2012) and endline (2016): baseline MMR=342 maternal deaths/75,675 live births*100,000; 2016 MMR=222 maternal deaths/87,094 live births*100,000.

c Zambia MMRs are adjusted estimates using General Growth Balance method for compensating underreporting of all deaths to WRA in the previous 12 months and applying the proportion of deaths among WRA that are due to maternal causes to derive maternal deaths; population live births were adjusted using P/F ratios estimated from the lifetime fertility of women of reproductive age. Adjusted baseline MMR=200 maternal deaths/41,665 live births; adjusted endline MMR=135/47,509 live births.

d Uganda cause-specific MMRs are direct estimates using population maternal deaths of a specific cause divided by total number of population live births. Zambia cause-specific MMRs are adjusted estimates using General Growth Balance method for adjusting all deaths to WRA and applying the proportion of deaths among WRA that are due to maternal causes to derive maternal deaths; crude percent distribution by cause is applied to the adjusted maternal deaths to derive adjusted cause-specific MMRs.

e Uganda time-of-death MMRs are direct estimates using population maternal deaths while pregnant (antepartum), during delivery or first 24 hours postpartum, and up to 42 days postpartum divided by total number of population live births. Zambia MMRs are adjusted estimates using General Growth Balance method for adjusting all deaths to WRA and applying the proportion of deaths among WRA that are due to maternal causes to derive maternal death; crude percent distribution by timing of death is applied to the adjusted maternal deaths to derive adjusted cause-specific MMRs.

In SMGL-supported districts, the reduction in maternal mortality was largely driven by declines in direct obstetric causes.

## DISCUSSION

Over a 5-year period, the SMGL initiative implemented a comprehensive health systems strengthening approach that focused on making rapid improvement in availability of, and access to, facility delivery and EmONC services. The increase in availability of services, together with community-level demand generation, was associated with a greater proportion of facility deliveries and improvement in health outcomes. The respective 44% and 41% declines in population-based maternal mortality in Uganda and Zambia learning districts attest to the success of the SMGL initiative in achieving its central goal. The magnitude of the reductions in maternal mortality within SMGL-supported districts during the short 5-year period was unprecedented in sub-Saharan Africa and is comparable with the decline achieved globally in 25 years.^1^ Between 2012 to 2016, the average annual decline in MMR in the project-supported districts was approximately 11% per year, compared with WHO's estimated annual reduction rate of 2.5% per year for sub-Saharan Africa and reductions of approximately 3% per year at the national levels documented in both countries' Demographic and Health Surveys.^21^^,^^40^

In both Uganda and Zambia SMGL-supported districts, declines in maternal mortality were significant during delivery and immediately postpartum (down by 72% in Uganda and 46% in Zambia), when SMGL interventions that focused on the intrapartum period would be expected to have their greatest impact. Evidence from Uganda, where population data were collected at the end of Phase 1, shows that two-thirds of the decline in maternal mortality in the SMGL-supported districts was achieved after the first “proof-of-concept” year, during which SMGL interventions were rapidly scaled up and implemented most intensively.^41^ Smaller but sustained declines in the subsequent 4 years demonstrate that Uganda SMGL-supported districts expanded the gains in maternal mortality reduction during Phase 2.

In Uganda and Zambia SMGL-supported districts, declines in maternal mortality were significant during delivery and immediately postpartum, when SMGL interventions that focused on the intrapartum period would be expected to have their greatest impact.

In addition to the reductions achieved in population-level maternal mortality, substantial gains were found for most other maternal and perinatal health indicators. This was likely related to the facility delivery rates increasing substantially and at a similar magnitude in Uganda and Zambia (47% and 44%, respectively). Nevertheless, Uganda's endline institutional delivery rate of 66.8% still leaves considerable room for continued improvement, with almost a third of deliveries still taking place outside a health facility. Models estimating potential lives saved indicate that about half of maternal and neonatal deaths and a third of stillbirths could be averted through scaling up maternal and newborn health interventions.^9^^,^^42^ In both countries, the number of CEmONC facilities increased, as did the delivery rates in higher-level EmONC facilities and the number of major direct obstetric complications treated. Both countries reported a significant increase in the population cesarean delivery rate. Uganda's cesarean delivery rate increased to 9%, which was well within the 5% to 15% range recommended by WHO. Although Zambia's cesarean delivery rate improved to 4.8%, still below the WHO recommendation, it had increased by 79% from SMGL baseline rate of 2.7%, indicating further improvement was possible. Greater utilization of adequately staffed and equipped health facilities in SMGL-supported districts and improved access to lifesaving interventions for mothers and their infants undoubtedly were instrumental in achieving and maintaining lower facility MMRs.

In both countries, the number of CEmONC facilities increased, as did the delivery rates in higher-level EmONC facilities and the number of major direct obstetric complications treated.

Intervention-specific data were not available to assess the individual impact of SMGL interventions. However, population-level data indicated that maternal deaths due to obstetric hemorrhage and obstructed labor, in general, declined significantly in both countries, as did obstetric hemorrhage at the facility level in both countries. These findings are consistent with the reported increase in use of active management of the third stage of labor, manual removal of placenta, removal of retained products, availability of blood transfusions, and obstetric surgery.

SMGL interventions were also associated with improved perinatal outcomes. The increase in the number of deliveries in EmONC facilities in both countries means that more obstetric and neonatal emergencies received appropriate care in a well-equipped facility where providers had been trained in and applied neonatal resuscitation techniques. SMGL facilities reported large increases in performance of neonatal resuscitation in the 3 months prior to each HFA (about 2.5-fold increase between baseline and endline). Other SMGL interventions that improved newborn outcomes included support for essential newborn care, early and exclusive breastfeeding, infection control practices, thermal care around the time of birth for all neonates, and kangaroo mother care for preterm babies.

Over the course of the SMGL initiative, facility perinatal mortality declined significantly, from 22.4 to 14.3 per 1,000 births in Uganda and from 37.9 to 28.2 per 1,000 births in Zambia. These reductions were driven by declines in the SBR, a finding that is consistent with the reported improved monitoring and care during labor and delivery; improved case management of obstetric complications; better access to emergency obstetric care, including obstetric surgeries; and increased focus on newborn care at birth, including neonatal resuscitation. In Uganda, where stillbirths that occurred during labor and delivery were enumerated separately, the intrapartum SBR reduction of 36% was the biggest driver of overall declines in the total SBR. Significant declines in the total SBR were documented in Zambia (36% by the end of the initiative) as well, although data could not be disaggregated by timing of stillbirth. Given the generally slow global progress toward reducing perinatal mortality and the particular lack of visibility of the burden of stillbirths,^43^ the reductions documented by SMGL represent an important achievement.

In Uganda, where stillbirths that occurred during labor and delivery were enumerated separately, the intrapartum SBR reduction of 36% was the biggest driver of overall declines in the total SBR.

However, predischarge NMRs measured by the SMGL facility monitoring did not change significantly in either country. The risk of neonatal death occurring during the early neonatal period—within approximately 24 to 48 hours of delivery—is very high across a range of countries, with an estimated 36% of neonatal deaths occurring on the first day of birth.^44^ In both Uganda and Zambia, predischarge NMRs remained unchanged and relatively high at SMGL endline—7.6 per 1,000 live births in Uganda and 8.7 per 1,000 live births in Zambia. The lack of NMR reduction indicates a compelling need for further investments in basic equipment and supplies for supportive care, such as oxygen, nasogastric feeding, and intravenous fluids; neonatal special or intensive care units; and training of clinical staff to help vulnerable babies survive adverse neonatal health conditions and/or complicated deliveries. It is possible that changes in reporting may have played a role in lack of change in the predischarge NMR, as increased training on neonatal resuscitation—conducted as part of the SMGL initiative—may have sensitized facility staff to a common misclassification of newborns as stillbirths if they did not initiate spontaneous breathing.^45^

In both Uganda and Zambia, predischarge NMRs remained unchanged and relatively high at SMGL endline.

Similar to improving maternal survival, targeting interventions to increase early neonatal survival requires adequate documentation of the number and rates of perinatal deaths as well as a systematic review of this information, including causes and contextual factors that may have contributed to these deaths. Because we were unable to obtain this level of documentation from SMGL facility registers, making it impossible to track changes in cause-specific mortality, we could not determine whether the SMGL initiative was associated with reduction in any specific underlying causes. For example, it is possible that individual causes such as birth asphyxia, a cause that was particularly targeted through training and mentoring of delivery providers, could have declined significantly in Uganda even if the overall 10% decline in the NMR did not reach statistical significance. Nevertheless, it remains clear that SMGL efforts to reduce neonatal mortality did not have the desired level of impact and that further efforts targeting neonatal survival need to be accelerated in the SMGL-supported districts. Future efforts to rapidly reduce maternal and neonatal mortality may be better positioned to address this continuing problem by taking stock of the lessons learned from the SMGL clinical interventions and using the findings to improve newborn care and by continuing to document perinatal outcomes with the new monitoring tools and procedures that were introduced in SMGL-supported facilities.

### Limitations

Limitations of the SMGL approaches in communities and facilities generally stemmed from: (1) the potential for increased demand for facility delivery to outpace the district health systems' capacity to deliver quality facility services, despite intense efforts to improve and expand facilities and staffing; (2) uneven distribution and coverage of EmONC services, resulting in continued disparities in access to services; (3) remaining gaps in quality of care in facilities; and (4) a rapid launch and ramping up of activities with gaps in funding availability, particularly after SMGL's intensive Phase 1. This proved challenging for the coordination, continuity, and sustainability of SMGL intervention and evaluation approaches.

Although extensive, the monitoring and evaluation methods implemented for tracking SMGL outcomes also had important limitations. In general, data quality and completeness of facility- and population-based data increased in both countries since one of the goals of the initiative was to improve health information systems and data-driven decision making at the district level. The SMGL initiative used several strategies to ensure data quality: (1) training and mentoring of facility staff to improve quality of information recorded in source registers; (2) recruitment of SMGL M&E officers and training in data collection, data entry, reviewing, and submitting data; and (3) development and use of data collection instructions, training manuals, and indicator reference sheets. While these measures generally ensured consistency in data quality during the initiative, the more accurate indicators were not strictly comparable with those measured during the baseline period. There are also differences in measurements between Uganda and Zambia, as each country used existing data systems and infrastructure to devise its own independent data-collection approach, making cross-country comparisons more difficult.

Ascertainment of the numbers and causes of maternal deaths before SMGL implementation was particularly challenging in both countries, which may have resulted in an underestimation of the MMR decline. The introduction of MDSR in Uganda in 2013 greatly improved reporting of maternal deaths in communities and facilities afterward. Similarly, the facility-based MDSR, introduced in 2015 in Zambia, led to increased detection and a higher MMR in the following year. The SMGL census population, birth, and mortality data in Zambia, in particular, were heavily underreported at baseline, when compared to external sources, and required complex adjustment factors.^21^^,^^34^ As a result, the maternal mortality at baseline may have been higher and MMR declines in both countries may be underestimated.

The introduction of MDSR in Uganda and facility-based MDSR in Zambia greatly improved identification and reporting of maternal deaths in communities and facilities.

Facility-based maternal and neonatal mortality rates are also prone to selection bias, as they include only a subset of the population who accessed obstetric care services and may not necessarily reside in the districts where these facilities are located. In large referral hospitals, such as Fort Portal Regional Referral Hospital in Uganda and Mansa General Hospital in Zambia, for example, about one-third of maternal deaths in 2016 were among patients referred from districts outside SMGL coverage.

Throughout the SMGL initiative, the number and rates of stillbirths and predischarge neonatal deaths among babies weighing ≥1000 grams were monitored from data recorded in maternity registers. In contrast to SMGL's monitoring of maternal deaths, identification of perinatal deaths was not enhanced through triangulation of multiple data sources, audits were less widespread, and underlying causes were not consistently reported. Additionally, individual-level maternal and delivery characteristics, Apgar scores, and birthweight were available only in Uganda, whereas only aggregate numbers of deaths were available in Zambia. Individual-level data on outcomes of neonatal resuscitation were not available in either country.

Despite the limitations in SMGL approaches and M&E methods, significant improvements occurred in most outcomes in both countries. However, the main effects of the SMGL initiative were captured by comparing outcomes in the pilot districts before and after SMGL implementation, without a control group in nonintervention districts. This was due, in part, to the rapid launch of SMGL interventions (within a couple of months) throughout the pilot districts and the inability of the team to establish an appropriate control group within that time period. Each country implemented district-level interventions with varying scope, intensity, and M&E methods. As the interventions were not evaluated independently, it is impossible to determine the relative impact of any individual intervention. The before-and-after evaluation approach also introduced inherent limitations in the ability to attribute positive health outcomes to the SMGL interventions.

## CONCLUSIONS

Following the introduction of the SMGL model, maternal mortality declined significantly in 8 learning districts in Uganda and Zambia This decline is likely due to parallel improvements of supply and demand for obstetric and newborn services coupled with improved quality of care at health facilities and improved coordination and health management throughout the districts. Although the implementation and emphasis of SMGL interventions were not identical in each district, maternal health outcomes in SMGL-supported districts and facilities improved in both countries.

The 44% and 41% declines in maternal mortality in SMGL-supported districts in Uganda and Zambia, respectively, and the more modest but significant decreases in perinatal mortality, were accomplished through a comprehensive district systems strengthening approach that led to reductions in the 3 delays that contribute to maternal and neonatal deaths. Maternal mortality reductions of this magnitude over a 5-year period demonstrate that it is possible to greatly accelerate progress in saving mothers' lives. Newborn lives, however, continue to require more sustained attention. The lessons learned from the SMGL initiative can inform policy makers and program managers in other low- and middle-income settings, where similar approaches can be used to rapidly reduce maternal and perinatal mortality.

## Notes

**Peer Reviewed**

**Funding:** Saving Mothers, Giving Life initiative implementation was primarily funded by the Office of the Global AIDS Coordinator, U.S. Agency for International Development, U.S. Centers for Disease Control and Prevention (Cooperative agreements GPS003057 and GPS002918), Merck for Mothers, and Every Mother Counts. The funding agencies had no influence or control over the content of this article.
